# Comparing Habitat Suitability and Connectivity Modeling Methods for Conserving Pronghorn Migrations

**DOI:** 10.1371/journal.pone.0049390

**Published:** 2012-11-16

**Authors:** Erin E. Poor, Colby Loucks, Andrew Jakes, Dean L. Urban

**Affiliations:** 1 Conservation Science Program, World Wildlife Fund, Washington D. C., United States of America; 2 Faculty of Environmental Design, University of Calgary, Calgary, Canada; 3 Nicholas School of the Environment, Duke University, Durham, North Carolina, United States of America; Université de Sherbrooke, Canada

## Abstract

Terrestrial long-distance migrations are declining globally: in North America, nearly 75% have been lost. Yet there has been limited research comparing habitat suitability and connectivity models to identify migration corridors across increasingly fragmented landscapes. Here we use pronghorn (*Antilocapra americana*) migrations in prairie habitat to compare two types of models that identify habitat suitability: maximum entropy (Maxent) and expert-based (Analytic Hierarchy Process). We used distance to wells, distance to water, NDVI, land cover, distance to roads, terrain shape and fence presence to parameterize the models. We then used the output of these models as cost surfaces to compare two common connectivity models, least-cost modeling (LCM) and circuit theory. Using pronghorn movement data from spring and fall migrations, we identified potential migration corridors by combining each habitat suitability model with each connectivity model. The best performing model combination was Maxent with LCM corridors across both seasons. Maxent out-performed expert-based habitat suitability models for both spring and fall migrations. However, expert-based corridors can perform relatively well and are a cost-effective alternative if species location data are unavailable. Corridors created using LCM out-performed circuit theory, as measured by the number of pronghorn GPS locations present within the corridors. We suggest the use of a tiered approach using different corridor widths for prioritizing conservation and mitigation actions, such as fence removal or conservation easements.

## Introduction

Migration—seasonal round trip movement between discrete areas unused at other times of the year [1]—is an important adaptation to avoid predation, periods of low resources, or severe conditions for many different taxa including birds, cetaceans, insects, rodents, frogs and ungulates. Hence, disruption of migration routes by denying access to the route or creating barriers within the routes is often followed by a population decline [2]. Long-distance terrestrial migrations are in steep decline globally [3]–[5]. Afro-palearctic migrant birds have shown severe population declines for the past three decades, possibly due to habitat loss [6], salmon in the western United States have experienced migration timing disruption and increased mortality due to dams [7], [8] and ungulates the world over face disrupted migration routes due to roads, fencing and other anthropogenic landscape features [4].

Identification of migratory routes is crucial to advance our overall understanding of migration and to catalyze actions to conserve them, as threats to migrations such as overhunting, habitat loss and fencing, continue to increase [4]. Yet, conservation efforts have often overlooked migration routes themselves. For example, globally, protected areas cover just five of 24 surveyed large ungulate migrations worldwide, primarily in Asia and southern Africa [4]. This lack of protection may in part be due to our current lack of knowledge of long-distance migrations [9], [5] although its significance in sustaining robust ungulate populations is clear [10], [1], [11], [12].

Temperate grasslands are one of the most imperiled ecosystems today [13], with only 4.6% of global temperate grasslands under protection of national or international laws, regulations and agreements [14]. Consequently, ungulate populations in temperate grasslands may be more vulnerable to declines [15] as they are utilized disproportionally by human populations. In addition, populations at the periphery of a species' range have limited abundances due to marginalized species specific abiotic and biotic conditions [16], [17]. As such, a higher percentage of these individuals may engage in long-distance migrations to encounter suitable conditions.

Conservation of migration routes is of particular interest in the northern plains of North America, where only 9% of native tall grass prairies and 43% of grasslands, savannas and shrublands remain [18]. Seventy-five percent of long-distance migrations, notably those of bison (*Bos bison*) and pronghorn (*Antilocapra americana*), have been lost largely due to anthropogenic factors [1]. Pronghorn, the sole extant member of the Antilocapridae family, have lost 64% of their range; the third highest percent of range loss among North American ungulates and carnivores combined [18]. The northern plains of North America, representing the northern edge of the pronghorn range, are fragmented as infrastructure associated with a growing population expands [19], [20]. Roads and fences pose physical barriers to ungulate movement and are of growing concern to ecologists [21], [22]. To conserve these ungulate migrations their migration routes first need to be identified. However, to date, there have been few studies analyzing the physical landscape features that are associated with pronghorn movements *during* migrations.

In this study, we use two years of pronghorn migration data to compare two habitat suitability modeling (HSM) methods, maximum entropy (Maxent) and expert-based Analytical Hierarchy Process (AHP) and two connectivity modeling methods, least-cost modeling and circuit theory, to assess their ability to predict long-distance seasonal pronghorn migrations. For both the spring and fall migrations, we assess the performance of HSM and connectivity modeling techniques, provide suggestions for improving corridor predictions, and suggest a conservation approach utilizing these corridors.

## Methods

### Study Area

**Figure 1:**
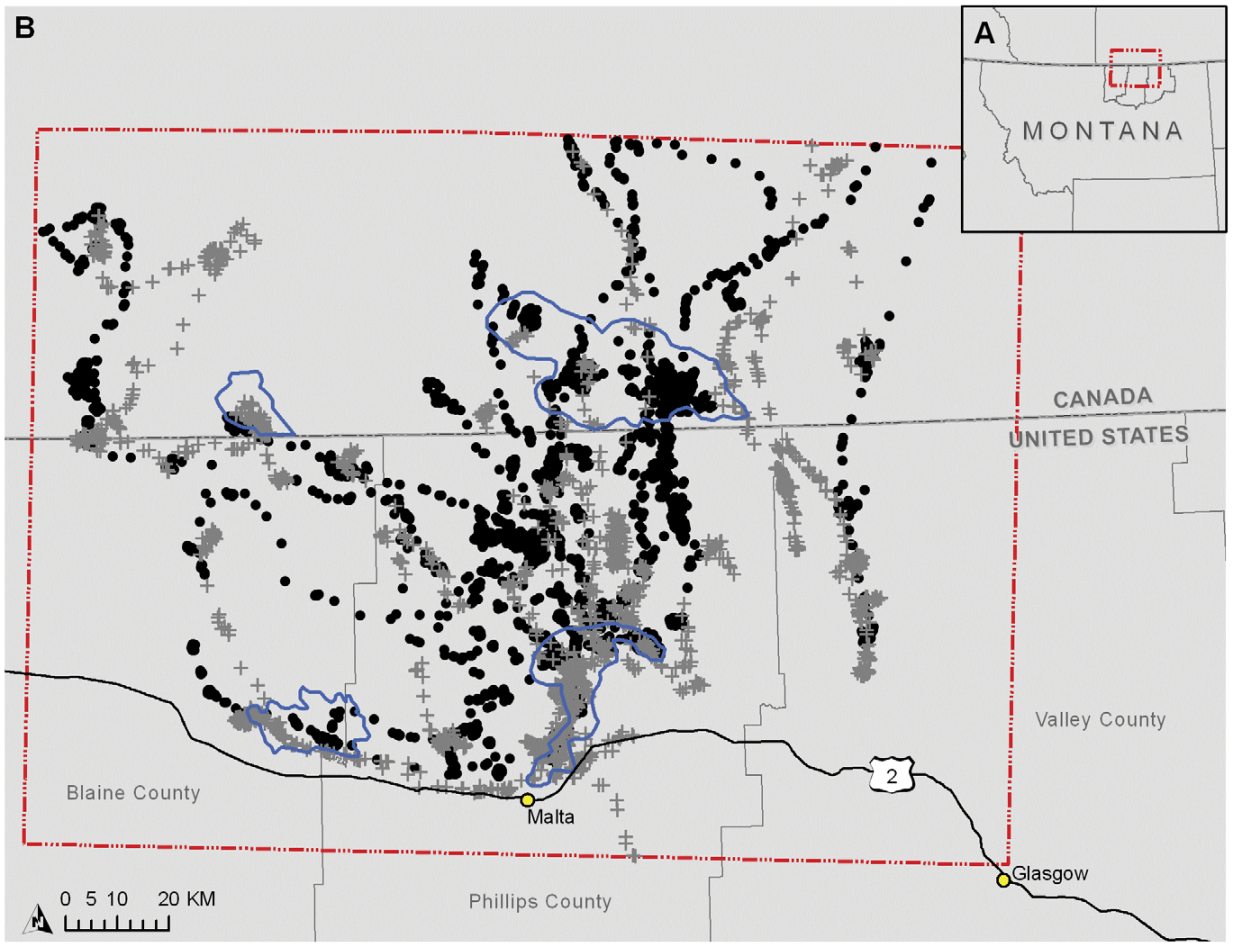
Study area and migration locations. Location of the study area in Montana and Saskatchewan (A) and pronghorn migrations for the spring of 2008 and 2009 (circles) and the fall of 2008 and 2009 (crosses) and the habitat patches (outlined in black) (B).

The study area included parts of Blaine, Valley and Phillips counties in Montana and the south-central portion of Saskatchewan (Figure 1). We only had access to land ownership data for Montana, where private lands made up the largest percentage of land ownership. Federal and state-owned lands were the second and third most abundant types of ownership, respectively (Table S1).

Major human activity within the study area includes dry crop and irrigated farming near riparian zones and natural gas drilling, which has occurred since the 1930's. There are large supplies of natural gas on both sides of the border. Paved roads are sparse, but major roads include US Highway 2 and Highway 191. The area is dotted with smaller towns (100–500 people), the largest being Malta with ∼2000 people. Fences follow all major roads on both sides and also act as boundaries between private and public properties. Most ranches in the area are cattle ranches but in years past sheep production was a major industry. Hence many private ranches still have sheep or woven wire fence as replacing fencing is costly.

Over half the study area iss grassland (56%) and over a quarter is in agricultural production (26%) (Table S2). Native grassland species include spear grass (*Stipa comate*), June grass (*Koeleria cristata*), Western wheatgrass (*Agropyron smithii*) and blue grama (*Bouteloua gracilis*). Evergreen shrubs, which provide year-round foraging opportunities to pronghorn, include silver sagebrush (*Artemisia cana*), big sagebrush (*Artemisia tridentate*) and pasture sagewort (*Artemisia frigid*). Other vegetation in the area includes western snowberry (S*ymphoricarpos occidentalis*) and prickly pear cactus (*Opuntia polyacantha*). Croplands in the area include alfalfa, hay, wheat, lentils and peas.

### Pronghorn Movement Data

Wildlife capture and handling professionals at Pathfinder Helicopters collared pronghorn in January 2008 and 2009 within the Bowdoin natural gas field (Figure 1), as part of a larger pronghorn study across northern Montana. This pronghorn herd has used these wintering grounds for generations, and all pronghorn were collared in this same area. We obtained wildlife capture and handling permit #11-2007 from the Montana Fish, Wildlife & Parks Institutional Animal Care and Use Committee (IACUC). In 2008, we fitted 22 female pronghorn with Lotek, Inc. 3300 GPS collars with remote-release mechanisms. In 2009, we captured an additional 20 female pronghorn. Because we wanted to record a full years migration (for both 2008 and 2009), we captured the pronghorn at the end of their migration routes. For the safety of the pronghorn, it is recommended to capture them in the winter as they cannot run as fast in the snow, decreasing the likelihood of breaking their legs. Pronghorn do migrate between Canada and the United States both east and west of our collaring location and so our area of study is a subset of the entire pronghorn migration in this part North America. We retrieved collars during winter using telemetry equipment.

We identify individuals that migrated >50 km for both seasons as ‘long-distance’ migrants. To ensure we captured only long-distance migration movements, we selected dates of migratory periods by mapping and graphically identifying natural break points in the daily movements of individuals [23], [24]. For migrating animals, these breaks were clear, indicated by a sudden large increase in daily movement. On average, daily movements during migration were longer than those during non-migratory periods (6,973 m vs. 4,827 m, respectively). To increase sample size for the analysis, we pooled the 2008 and 2009 data. Over both years, 17 of the 42 collared individuals migrated in the spring and 18 migrated in the fall (Figure 1). The remaining individuals remained relatively close to where they were collared and were deemed ‘non-migratory’. These individuals either had overlapping seasonal home ranges, or did not migrate at least 50 km.

### Predictor Variables

Literature regarding pronghorn preferences during migration is limited. As such we selected seven variables that likely influence pronghorn habitat selection and long-distance migration movements. We included four natural environmental variables: distance to water, land cover, forage greenness and terrain shape (Text S1). Yoakum et al. [25] found that pronghorn spent more time in habitat that was within 1–4 miles of a water source, although forage moisture may influence water requirements [26]. Normalized difference vegetation index (NDVI) was used as a proxy for vegetation greenness and forage quality [27], [28]. Because open landscapes allow pronghorn to watch for predators, pronghorn prefer gently sloping terrain and generally avoid rugged areas [29]. However, they have been known to traverse steep slopes when seeking protection from severe weather or if preferred forage is present [30]–[32] (Text S1).

We also included three anthropogenic variables in the models: distance to oil and gas wells, distance to roads and presence of fences (Text S1). We collared pronghorn on their winter range within the Bowdoin Natural Gas Field. This gas field has been in production since the 1920's at low densities (∼4 wells per township section). In 2008, an additional 1,250 wells were drilled and another 430 wells were replaced, thus doubling the number of wells in production. In Wyoming, development attributed to energy extraction negatively altered pronghorn habitat [33] and pronghorn avoided these areas of disturbance [22], [33]. The infrastructure development that often accompanies drilling, including roads, may compound negative effects of energy exploration on pronghorn movement [33]. Pronghorn display higher vigilance and spent less time foraging along roads regardless of traffic level, indicating that pronghorn perceive roads as a risk or a predation threat [34]. In our study area, fences are often associated with roads. Pronghorn often pass underneath fences but [35] when they do jump, they may get tangled in the top wires of most fence types [21]. We modeled fencing in Montana by making a series of assumptions about where fences are located along land ownership boundaries and roads. We then merged and dissolved land tenure and roads datasets according to the assumptions, which resulted in a GIS line dataset representing fences (Text S1). To assess the accuracy of modeled fence data, we collected a total of 1,788 GPS ground truth points of fences. We collected these mainly in the southeast part of the study area, extending to the towns of Coburg and Turner in the west and to the Canadian border in the north due to time and funding constraints. To account for potential positional error, we buffered the ground truth points by 15 m. If modeled fencing fell within this buffered area we counted it as an accurate fence. The modeled fence layer was >82% accurate using these methods (E.E. Poor and A. Jakes, World Wildlife Fund, unpublished data).

### Habitat Suitability Models

Habitat suitability modeling (HSM) techniques have been central to refining species distribution maps [36], [37], identifying movement pathways [38], [39] and prioritizing areas for habitat restoration or species reintroduction [38], [40]. Maximum entropy, or Maxent, identifies habitat using only presence locations [41], [42], which is compatible with our GPS data associated with pronghorn movements. We chose to use Maxent as compared, for example, to developing a Resource Selection Function (RSF), because many of the same assumptions (the use of pseudo-absence locations, that habitat remains constant across conditions), are present in both models. However, Maxent is a much faster model to develop, and it provides tools for statistical interpretation of the outputs and an ArcGIS-compatible output. Another method that has maintained popularity due to its simplicity is the expert-based modeling technique, or Analytic Hierarchy Process (AHP), which allows habitat modeling when empirical data are not available [43]. This approach can be inexpensive but can introduce uncertainty from the expert's perception or memory of the species or landscape in question [44], [42]. Results for expert-based habitat modeling generally improve when paired with literature review [44] and when long-term local experts are used [43]. One advantage of both AHP and Maxent is that neither requires many species location data, which can be expensive and time consuming to collect. While other techniques are available (such as generalized linear models, Genetic Algorithm for Rule-set Production and bioclimatic envelope models), we chose to compare these two methods because they are commonly used, easily replicated, and relatively intuitive to understand. This is especially important in communicating the results to private landowners who will be critical to the conservation of pronghorn migration routes.

The Maxent approach can be best understood as a combination of three ideas. First, the model fits an empirical density distribution to observed presences in terms of the predictor variables [41], [42], [45]. The distribution is described piecewise, using ‘features’ of the predictors (e.g., linear, quadratic, ramps, etc.). Second, the solution is approximated using maximum entropy principles, which ensures the simplest (in terms of parsimony) model consistent with the data, and imposes the fewest constraints on the model as possible [45]. With presence-only data, presence locations are chosen at random and compared to randomly chosen pseudo-absence sites [46]. Third, the model, implemented in the Maxent software package [41], provides results in a format similar to a GLM in that they are rescaled to mimic logistic regression results (i.e., scaled on [0, 1]) and the performance is measured in terms of receiver operator characteristics (ROC) curves [47]. In this, ‘true positives’ are cases where an actual presence point is modeled as ‘habitat’ (model sensitivity in ROC terms). Because the model does not require true absences, the ‘true negatives’ are created by counting the total proportion of the study area predicted to be habitat (analogous to model specificity in ROC terms) [36]. The goal is to maximize true positives while minimizing the predicted habitat area [48]. In Maxent, results are reported in terms of area under the curve, or AUC, where .5 represents a model no better than random at predicting the species distribution, and values closer to 1 represent better model prediction. The software provides additional information to help interpret the contribution of each environmental variable to the habitat model. In this, a jack-knifing procedure tallies the explanatory power of each variable used individually as well as the loss of explanatory power as each variable is withheld from the full model. We log transformed the Maxent output [49] and inverted the resulting HSM to create a cost or resistance surface.

There were a total of 5,364 pronghorn location points for the spring Maxent model and 3,486 for the fall model. We randomly held back 25% of the points for model testing and used the remaining 75% for model training. Doing so allows calculation of percent contributions of each variable. Training data is used for creating the models and is presence only. Because we collared pronghorn within an oil and gas field, the distance to wells variable was not used in this model.

The AHP is a tool used for multiple-criterion decision making in which, when used to model suitable habitat, experts conduct pair-wise comparisons of environmental variables based on observed or predicted habitat suitability [50], [43]. We included all variables in the AHP model to gain an understanding of how wells are perceived among experts. We focused efforts on recruiting experts most familiar with northern pronghorn (*A. americana americana*). Experts filled out a survey weighting each variable against every other variable and weighting each category within each variable against every other category within the same variable [50] (Table 1). We used a scale of 1–9, where 1 represented equal importance, increasing in difference numerically to 9, which represented the greatest importance of the variable or category in determining pronghorn presence, compared to the other variable or category [50]. We sent surveys to 26 individuals and received 12 completed surveys, though one expert only completed the fall season, resulting in 12 surveys for the fall migration and 11 for the spring with which to create the HSM. Respondents included five individuals representing federal, state or provincial agencies, two representing NGOs, one representing a private consulting firm and four graduate students studying pronghorn. Experience ranged from three years to more than a decade. We then calculated relative weights for each variable and for categories within the variables and used these as coefficients in creation of the HSM [43], which was then inverted (subtracted from one and rescaled to reflect positive values) within ArcGIS to form a cost surface.

**Table 1:** Variables included in pronghorn habitat suitability modeling in Montana and Saskatchewan, including attributes used by experts in the Analytical Hierarchy Process to assess importance of the categories used within variables.

Variable	Description	Attributes	Source
Distance to wells	Distance at which pronghorn avoid wells	0–150 m, 150–1000 m, >1000 m	Saskatchewan Ministry of Environment; DNRC Montana Board of Oil and Gas 2009
Distance to water	Distance at which pronghorn remain to water sources	0–1000 m, 1000–10000 m, >10000 m	2000 National Hydrography Dataset Waterbody Features; Atlas of Canada 1,000,000 National Frameworks Data Hydrology-Drainage Network
Distance to roads	Distance at which pronghorn avoid roads	0–300 m, 300–1000 m, >1000 m	2000 TIGER, 2004 Saskatchewan Enhanced SURN Dataset
Normalized difference vegetation index	Relative greenness	>0.6, 0.3–0.6, 0.2–0.3, <0.2	Land Processes Distributed Active Archive Center (LP DAAC), located at the U.S. Geological Survey Earth Resources Observation and Science Center
Fence presence	Fence locations	Present, Absent	Modeled with Cadastral Database from Montana Department of Administration, Information Technology Services Division and Department of Revenue
Land cover	Land cover type	Water, Development, Shrublands-Grasslands, Wetlands-Riparian, Agriculture, Pasture and Perennial Crops	Montana Fish, Wildlife and Parks National Gap Analysis 2000; Land Cover for Agricultural Regions of Canada
Topographic position	Position of landscape relative to surrounding landscape	Canyon bottom, Flat-gentle slopes, Steep Slopes, Ridge top	Ministry of Economy, Trade, and Industry of Japan and U.S. NASA

### Connectivity Models

After suitable habitat is identified, models identifying areas of connectivity are developed. Some landscape connectivity theories are based in graph theory, an emerging tool in conservation planning [51]. Graphs, composed of habitat patches and habitat links (corridors) connecting the patches, may be used to illustrate the movement of populations and facilitate landscape connectivity analyses [52]. Currently there are two popular methods for implementing graph theory in corridor identification: least-cost modeling (LCM) [53], [40] and circuit theory [54]. In LCM, the path of least resistance between two points across a cost surface is identified [53]. This method has performed well but it is recognized that wildlife may not travel through the single path of least resistance and the distance traveled is likely greater [55]. As an alternative, an application of electrical engineering, circuit theory [54], allows identification of multiple paths of current flow between habitat patches [52]. The use of circuit theory as a corridor identification tool is relatively new but has been shown useful in modeling gene flow across landscapes [56], [57].

Both least-cost modeling and circuit theory implemented using Circuitscape version 3.5 [58], required the identification of ‘source’ and ’destination’ habitat patches. We set a threshold on the Maxent resistance surface of equal sensitivity and specificity, converted this to a raster and isolated these areas of ‘good’ habitat within ArcGIS. We identified the migration ‘start’ by overlapping the area where pronghorn were collared with areas of good habitat. End locations were identified by areas of high pronghorn density and good habitat. We used Grasslands National Park in Saskatchewan, the only federally protected area in our study area, as one of the end locations. During the spring migration three northern habitat patches acted as the ‘destinations’ from the ‘source’ patch where they were collared (Figure 1). We reversed this functionality for the fall migration as pronghorn travel southward from the northern patches (Figure 1). To create the LCM corridors, we calculated the cost distance from each patch to every cell and summed to determine the path of least resistance.

In contrast, circuit theory is based in Markovian random walk theory and describes every movement as a random choice with movement in every direction equally probable [54], [59]. The landscape then acts as an electrical-resistance surface or, inversely, as a conductance surface similar to a HSM, as the current travels outward to surrounding cells from the source patch [59]. The areas of least resistance or greatest conductance across the landscape are the most probable areas for movement [59]. The Maxent and AHP HSM were treated as resistance grids and each habitat patch as a focal region which alternatively acted as the current source.

For both models we identified corridors by implementing resistance thresholds based on the percent of most traversable habitat—areas on the landscape through which pronghorn move—and compared the resulting corridors. To gain an understanding of how corridor performance changes with changes in movement difficulty, we set thresholds on the cost surfaces to identify 1%, 5%, 10%, 15%, and 20% of the most traversable habitat using the Corridor Designer ArcGIS toolbox [60]. This resulted in five corridors of differing widths, allowing us to identify the area required to conserve the most pronghorn migration habitat and while also providing different options for conservation in terms of a tiered conservation approach. Circuitscape has not previously been used in this context, but we aimed to test its ability as a corridor identification tool in a relatively open landscape and to compare resulting corridors with the more common LCM method.

We projected all geographic information systems (GIS) data to NAD 1983 UTM Zone 13 north and we prepared the data in ArcGIS Desktop 9.3.1 (Esri 2009). Data resolution was 30 m for analysis, except in identifying Circuitscape corridors, where we resampled the data to 90 m due to processing constraints.

In total, we created four resistance surfaces; two for the spring season (AHP and Maxent) and two for the fall season (AHP and Maxent). Using these resistance surfaces, we created eight sets of corridors, four sets each for both seasons using LCM and Circuitscape connectivity modeling methods: AHP-LCM, AHP-Circuitscape, Maxent-Circuitscape and Maxent-LCM (Figures S1, S2). We assessed performance of the HSM and corridors using the resistance thresholds and comparing them to habitat area and the percent of pronghorn locations within them.

## Results

### Habitat Suitability Models

Distance to water, vegetation greenness (NDVI) and land cover were the most important parameters, with total relative contributions >85% for both seasons (Table 2). Distance to water was the highest contributor during the fall, while NDVI was the highest during the spring (Table 2) and both of these variables had the highest influence when included as the only variable in their respective models. In the fall, lower NDVI values predicted pronghorn presence while in the spring, higher NDVI values predicted presence. Topographic position and fencing had the lowest relative contribution for each season (Table 2). The AUC for spring and fall migration seasons for the Maxent HSM was 0.66 and 0.68, respectively.

**Table 2:** Relative contributions of Maxent and the Analytical Hierarchy Process (AHP) models of pronghorn suitable migration habitat in Montana and Saskatchewan.

	Maxent		AHP^1^	
Variable	Spring	Fall	Spring	Fall
Distance to wells	NA	NA	7.74	5.29
Distance to roads	11.7	6.4	8.1	11.6
Distance to water	21.3	45.1	6.04	8.1
Normalized Difference Vegetation Index	43	24.7	21.4	17.1
Fence Presence	0.2	0	17.05	21
Land cover	23.7	21.6	23.1	21.2
Topographic position	0	2.2	16.57	15.7

1 Analytic Hierarchy Process.

Among pronghorn experts, land cover was identified as the most important variable in determining pronghorn presence during both seasons, although the perceived importance of land cover category varied seasonally (Table 2). Vegetation greenness (NDVI) was also highly ranked for both seasons, with moderate NDVI classifications scoring the highest for both seasons. During the spring, distance to water was ranked the least important variable but at the category level, experts thought areas closer to water were more important than areas far from water. During fall migration, experts ranked distance to wells the lowest (Table 2). In both seasons, areas closest to wells and roads were ranked the least important areas in determining pronghorn presence. Areas without fencing and areas with flat-gentle slopes were considered the most important features for pronghorn presence within these variables for both spring and fall.

### Connectivity Models

#### Spring

**Figure 2:**
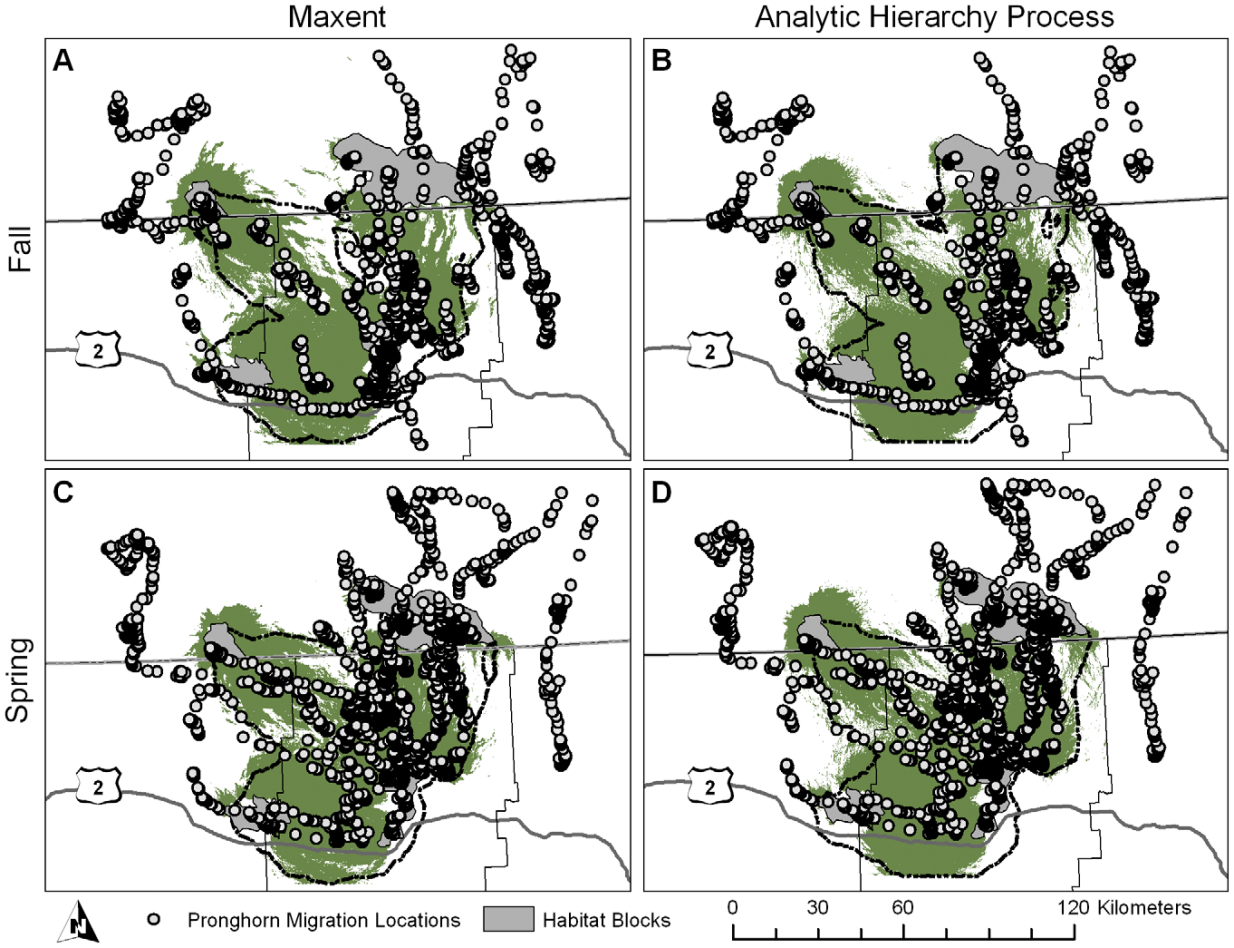
Corridors created using 10% threshold. Least-cost modeling (solid line) and Circuitscape (shaded area) pronghorn migration corridors in Montana and Saskatchewan created from the 10% most traversable habitat on Maxent and Analytic Hierarchy Process resistance surfaces for fall (A) and (B), respectively, and spring (C) and (D) migration seasons.

**Figure 3:**
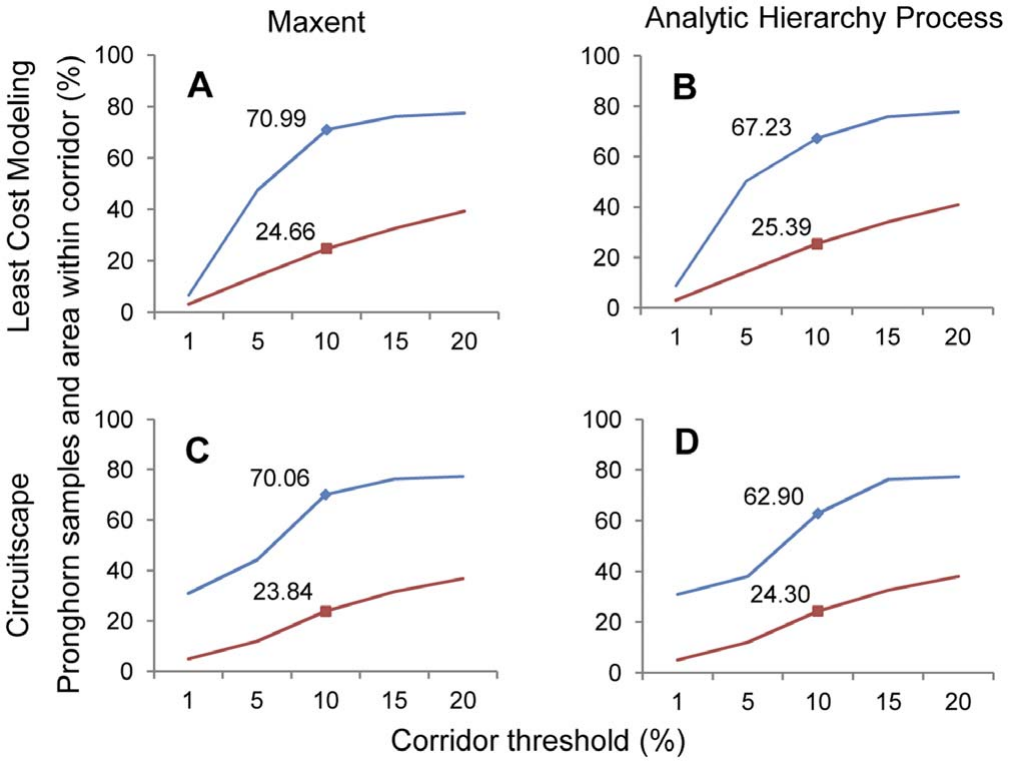
Pronghorn and area included within spring corridors. Percent of pronghorn locations and study area in Montana and Saskatchewan for the spring pronghorn connectivity models. Maxent resistance surface and least-cost modeling (LCM) (A), Analytic Hierarchy Process (AHP) resistance surface and LCM corridors (B), Maxent resistance surface and Circuitscape (C) and AHP resistance surface and the Circuitscape connectivity model (D).

In the spring, nearly all corridors reached an asymptote at the 10% threshold when considering the amount of area and number of pronghorn locations included within the corridors (Figure 2C, Figure 2D & Figure 3). The Maxent-LCM corridor contained nearly 71% of pronghorn points in nearly 25% of the study area (Figure 3A), making it the best corridor for spring with the most number of pronghorn locations in the least amount of area. On average, all corridors created using the Maxent resistance surface included a higher percentage of pronghorn (58% versus 57% with AHP resistance surfaces, respectively) within slightly less area than those created using AHP (Figures S1, S2, S3, S4). Least-cost modeling corridors included more pronghorn locations than Circuitscape corridors in most cases (Figure 3). In comparing the mean number of pronghorn locations within the 5–20% LCM and Circuitscape corridors, LCM corridors contained more pronghorn points on both Maxent (68%) and AHP (68%) surfaces than Circuitscape corridors did (67% on Maxent and 64% on AHP surfaces) (Figures S1, S2, S3, S4, S5).

There were on average 296 fixes recorded per individual during the spring migration (Tables S3, S4, S5, S6). In the spring, on average 21% of individual point locations were within the 1% corridors, 50% within the 5% corridors, and 72% within the 10% corridors. In most cases, entire individual movement pathways were not completely included in the 1% and 5% corridors. However, six individual pathways fell completely within the Maxent-LCP 10% corridor (Tables S3, S4, S5, S6, S7, S8 S9, S10).

#### Fall

**Figure 4:**
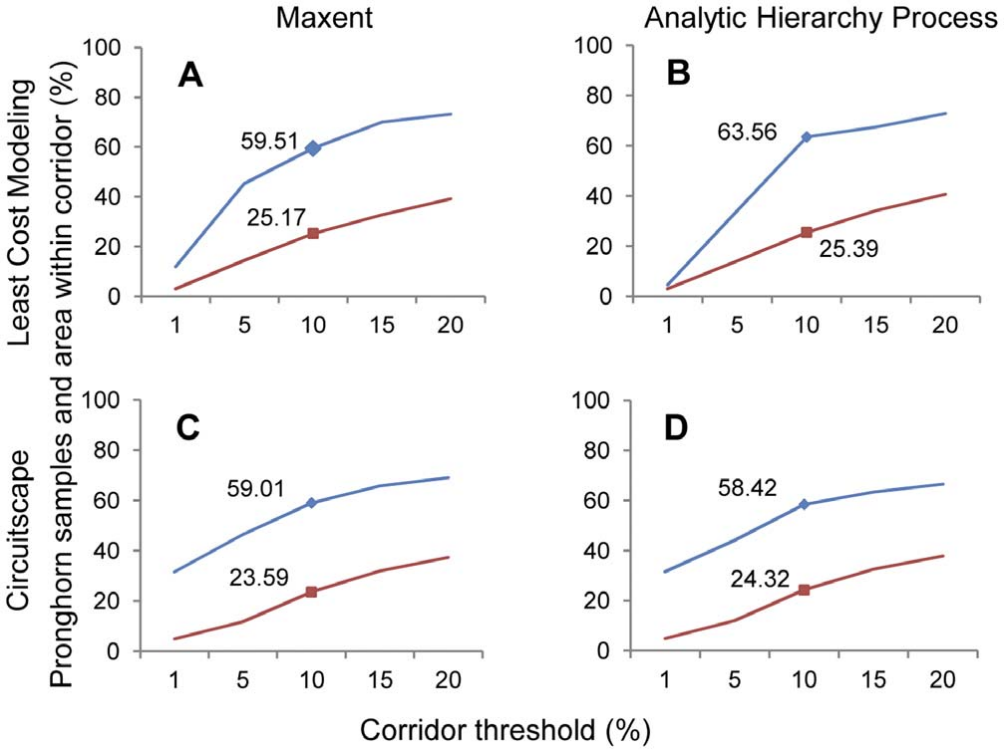
Pronghorn and area included within fall corridors. Percent of pronghorn locations and percent of study area within Montana and Saskatchewan for the fall pronghorn connectivity models. Maxent resistance surface and least-cost modeling (LCM) (A), Analytic Hierarchy Process (AHP) resistance surface and LCM corridors (B), Maxent resistance surface and Circuitscape (C) and AHP resistance surface and the Circuitscape connectivity model (D).

Overall, the fall corridors included fewer pronghorn in a similar area as spring corridors. We found similar asymptotic effects of the 10% corridors in the fall analyses (Figure 2A, Figure 2B & Figure 4). The 10% Maxent-LCM corridors contained 60% of the pronghorn in 25% of the study area (Figure 4A). As with spring, corridors created using the Maxent resistance surface contained, on average, a greater percentage of pronghorn locations (53% versus 51% on AHP) and a smaller amount of area (22% compared to 23%) (Figures S1, S2, S3, S4). However, the single best corridor for the season was the 10% AHP-LCM corridor, including 64% of pronghorn locations in 25% of the study area. Excluding the 1% corridors, LCM corridors included on average, a larger percent of pronghorn locations. Least-cost modeling corridors contained 63% of pronghorn on Maxent surfaces and 60% on AHP surfaces, whereas Circuitscape corridors contained 60% and 58% on Maxent and AHP, respectively.

On average, 183 fixes were recorded per individual during the fall migration. An average of 21% of individual point locations were include within the 1% corridors, 48% within 5% corridors and 68% within 10% corridors. In the CS corridors, entire pronghorn pathways were not included in corridors until the 15% threshold, whereas in LCP corridors, one individual's path fell completely within a 5% corridor. Five pronghorn pathways fell completely within the 10% Maxent-LCP corridor (Tables S3, S4, S5, S6, S7, S8, S9, S10). One individual did not fall within any corridor during spring or fall seasons.

## Discussion

Our results identify highly utilized migration routes between wintering and summering areas for pronghorn in Montana's northern prairie region. We show expert-based corridors (regardless of connectivity modeling method) can perform relatively well and are a cost-effective alternative if species location data are not available. Maxent-derived corridors may be a good choice if limited species location data is available. Overall, Maxent-LCM corridors connected the three habitat blocks and included more pronghorn locations than other corridor combinations. We recommend that these areas be given priority and evaluated for potential future conservation efforts.

### Habitat Preferences during Migration

Regardless of connectivity model used, we found corridors created with Maxent resistance surfaces contained more pronghorn locations in less area than those created using AHP. However, due to the use of stopover locations by our collared pronghorn, the Maxent output may be skewed toward estimating stopover habitat, instead of migration-only habitat. Since we were unaware of any published studies that identified habitat that pronghorn use during migration, we included these pronghorn locations as they were deemed critical to successful migrations. Although some bias is inherent in presence-only modeling [45], Maxent does not introduce external bias as found in expert-based models [44]. Rarely have expert-based models been used to describe behavior during migrations and it may have been difficult for our experts to rank variables solely on preferences during migration.

Overall, spring migration models performed slightly better than fall models. This may be due to the increased variability of fall movements, because pronghorn make unpredictable and exploratory movements as winter weather varies [61]. In the fall Maxent model, distance to water was identified as the single most important environmental variable. Although NDVI was ranked much lower in the fall season than the spring, it had a much higher percent contribution than the other variables. More predictable routes are taken in the spring as pronghorn follow greening high-quality forage to fawning grounds [62], as suggested by our analyses, indicating a greater proportion of locations in our corridors during the spring migration. Land cover, NDVI and distance to water were identified as the most important factors during spring migration suggesting pronghorn follow vegetation greenness or forage quality during migration. These results correspond with other studies investigating ungulate migration habitat [63], [64], but migration habitat preferences for this and other ungulate populations still remains a gap in migration literature.

Both experts and Maxent identified differences in pronghorn preferences between migration seasons. In the Maxent outputs, NDVI was identified as a key determinate of pronghorn location in the spring, while experts identified land cover as the key driver of pronghorn location in the spring. Distance to water was the primary determinate in the fall season in Maxent, and experts again identified land cover (though with a lower score than their spring ranking) as the key driver of pronghorn migration. The changes in percent contribution of each variable from one season to the next contributed to the differences in the corridors between seasons. These differences in the Maxent outputs arose from the differences in pronghorn positions on the landscape between the fall and spring seasons. Overall, fall pronghorn locations were more often found in areas near water even if these areas had a lower NDVI. In the spring, pronghorn were located more often in areas of high NDVI regardless of the distance to water. Because experts were not shown the pronghorn locations and hence did not take their locations into account when judging the variables, they could not pick out these larger differences in location preference between seasons. Additionally, experts were asked to rank the influence of oil and gas wells on pronghorn location, which may contribute to the seemingly larger percent contributions of the other variables in the Maxent outputs. Due to this, the Maxent-derived cost surfaces show greater differences between the seasons than do the expert-derived cost surfaces (Figure S1, S2, S3, S4, S5).

One shortcoming of Maxent is that it analyzes habitat on a cell-by-cell basis whereas experts can take a larger perspective in terms of time and space. This difference became apparent in the ranking of fence influence on pronghorn habitat. The experts ranked the presence of fences much higher than they were ranked using Maxent. Experts likely thought fences would inhibit movement during migration due to prior knowledge. Because we used a measure of fence presence/absence instead of a distance or density measure, a raster cell directly next to a fence may be marked as suitable habitat and unless a pronghorn location point fell directly on a fence, fences may have no effect on pronghorn presence within Maxent. Although fencing may inhibit pronghorn movement across a landscape, it may not have a large influence on what defines ‘suitable habitat’ for pronghorn at a local scale. In the future, using a measure of fence density across the region of interest is likely to be a more robust measure of fence influence.

### Migration Corridors

Although both least-cost modeling and Circuitscape can be used to identify connectivity, they may be best used to identify different types of connectivity. Least-cost modeling can be used to identify the path of least resistance across a landscape, whereas Circuitscape can identify the best area of flow across a landscape from a single location. Circuitscape has been recommended as a landscape connectivity tool. When used as such, no destination habitat block is required and connectivity across the entire landscape is identified from a source habitat block. When used as a corridor identification tool, multiple alternative pathways are identified from the source and destination habitat blocks and these may not connect to form a corridor. It is possible to use least-cost modeling as a landscape connectivity tool in the same way, but it is recommended as a corridor identification tool. A least-cost model identifies the single optimal pathway between two habitat patches and will result in a connected corridor between them. Although both tools have many uses, Circuitscape may be better used in situations such as identifying dispersal rates [59], and least-cost modeling may be best used to identify migration routes.

Despite being designed as a landscape connectivity tool, Circuitscape corridors performed relatively well in our study area. When comparing LCM to Circuitscape corridors created using the same habitat model, LCM generally resulted in corridors that contained more pronghorn locations often with less area, although overall differences were minimal. However, in other landscapes with more topographic features or are more highly fragmented, Circuitscape corridors may not result in wildlife corridors, but in areas where current is forced through a narrow space which increases the current flow [59]. In relatively open landscapes such as our study area, current is highest at the habitat patches as they are ‘connected’ to a current source and current dissipates outward in all directions across the landscape. When thresholds are selected, the habitat patches themselves are within the top threshold and patches may not be connected to each other through a ‘corridor’ until a higher percentage threshold is selected. In our study the Circuitscape corridors did not connect the habitat patches until the 10% threshold level (Figure 2, Figure 3).

Although our Maxent corridor models conflate migrating and stopover habitat, high quality vegetation stopover areas are critical for ungulates to follow during migrations [62], However, focusing conservation resources solely on protecting seasonal home ranges and stopover points would likely be insufficient if development continues between these areas. All necessary parts of a pronghorn's range (including seasonal home ranges and stopovers) should remain functionally connected, even if ‘connected’ means some development is allowed due to a species' ability to move through otherwise sub-par habitat.

**Figure 5:**
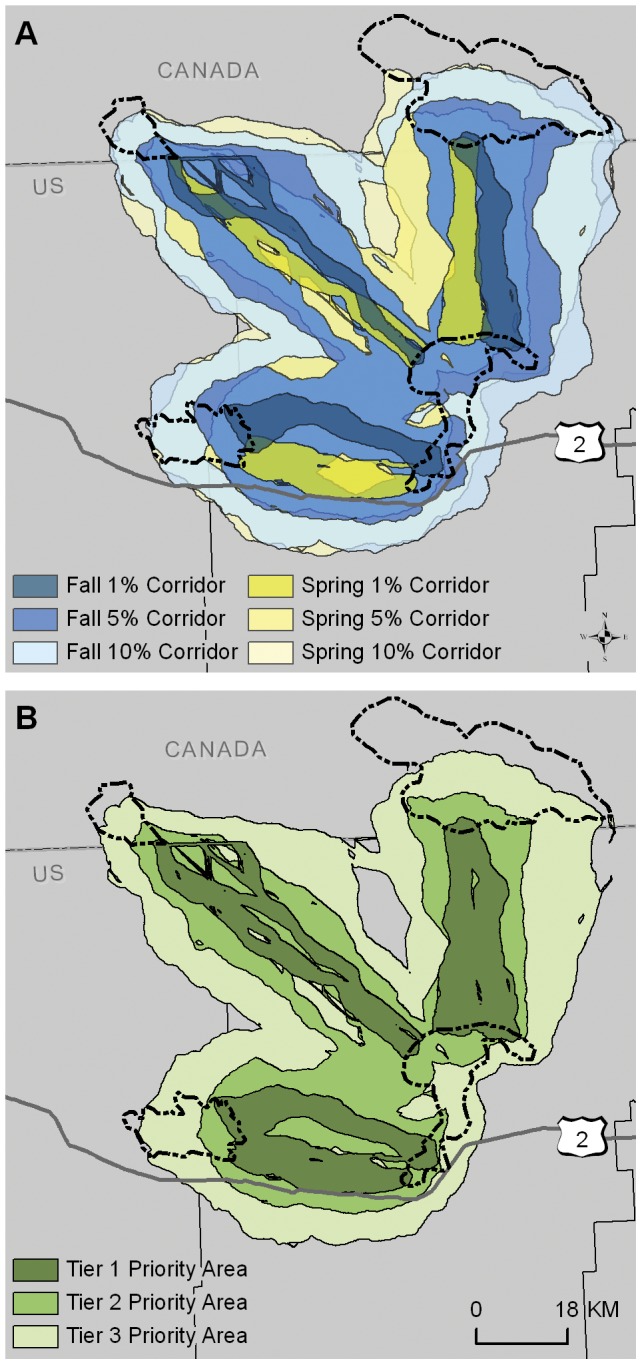
Top corridors and suggested Pronghorn Priority Areas. The top corridors identified (the least-cost model-Maxent 1%–15% corridors) for fall (blue) and spring (yellow)(A) and the resulting recommended tiered Pronghorn Priority Areas, created from the 1% corridors (Tier 1, dark green), 5% corridors (Tier 2, medium green) and 10% LCM-Maxent corridors (Tier 3, light green) (B).

To conserve both spring and fall migratory routes, stopovers and seasonal ranges, and make the best use of limited resources across vast landscapes, we suggest the use of a tiered approach for prioritizing corridor conservation. Tier 1 pronghorn priority areas should include those areas most important to the pronghorn migration, namely those created using the 1% thresholds which are included in either the fall or spring Maxent-LCM corridors (Figure 5). These areas have been identified as the most traversable habitat and are included in all larger corridors. Tier 2 priority areas (Figure 5) include the overlap between the spring and fall corridors created using the 5% threshold. Tier 3 conservation efforts should focus on the areas of overlap of the spring and fall 10% corridors (Figure 5). Using a tiered approach focuses on the most important areas for maintaining connectivity, prioritizing resources to provide the most impact. It should be noted that the corridor area defined by the percentage threshold depends on the boundary of the study area, and results are specific to our study area.

### Model Choice

This study is not an exhaustive comparison of AHP and Maxent or of habitat suitability and connectivity modeling methods in general. We compared only two habitat and two connectivity modeling methods using their recommended parameters [50], [44], [49]. We chose to compare these particular methods because they are often used in existing wildlife literature due to their ease and quickness of use. Additionally, we want our results to be easily interpretable for private land owners who make up a large proportion of our landscape. However, we recognize that the use of a resource selection function and a step selection function to determine habitat use and movement patterns could provide additional insight into the migration ecology of this pronghorn population. We recommend exploring these methods in future analyses.

Expert-based corridors and Maxent can both perform relatively well if there are no, or few, species data available. Alternatively, if detailed species data are available, recent work has shown the potential of utilization distribution (UD) methods, such as kernel density estimation [65] and Brownian bridge movement models, in delineating migration corridors [66], [67]. Our results may have been different using Brownian Bridge modeling, which allows estimation of the pathway each individual has taken during a period of movement. Brownian bridge modeling depends the on time and distance between successive species locations [66] whereas kernel density estimates do not utilize time between locations and assumes locations are temporally independent [67]. These approaches incorporate all animal movement data and allow movement routes to be estimated and averaged across individual animals. Pathways may have more or less uncertainty and be wide or narrow depending on the temporal scale of the available data. However, the uncertainty of these models decreases with more points collected at a higher fix-rate, which may be costly. Furthermore, such models are helpful only in estimating migration pathways already taken, not in predicting pathways where species data are unavailable. Had we collected fixes every few hours or sub-hourly, we could have identified fine scale movement pathways for individual pronghorn, with low uncertainty. We recognize the potential power and insight a Brownian bridge analysis can provide, but we unfortunately did not have access to data conducive to Brownian bridge movement modeling. Furthermore, because we were looking to predict where future members of this pronghorn herd may travel during migration, and to identify areas of the landscape that could be targeted for fence and road mitigation measures, we deemed the use of Circuitscape and least-cost corridor modeling appropriate.

### Potential Improvements

While our results are robust in comparing various methods for delineating corridors using these selected methods, we identify some factors that would improve future analyses. First, improvement of the fence data could increase the accuracy of the models. The fence data did not include permeability attributes, as some fencing types allow easier wildlife passage than others [21], nor did it incorporate land cover which may provide historical perspectives to the use of fencing. In addition, the model accounted for fences only on one side of maintained roads. Many roads have fences on both sides, likely causing increased resistance to movement. Further research is needed on the effects of potential barriers to ungulate habitat selection and migration as well as habitat selection within migration corridors and stopover sites.

Lastly, to increase sample size, we combined 2008 and 2009 pronghorn data. Annual fluctuations, especially during winter and at the northern limits of their range may dictate population dynamics and behavioral strategies. Individuals carry over effects from one season to the next [5] which can explain reproductive success, annual survival and general condition variability in individuals, ultimately influencing migrations in subsequent seasons [68]. Therefore long-distance migrations and potential corridors would ideally be analyzed on an annual basis to best identify corridor use and importance over time and how additional impediments to movement may influence future use by populations. However, as our study focused on testing habitat suitability and connectivity models, we felt combining the data would improve model comparison.

## Conclusions

In addition to conserving the land where migrations occur, conservation of a migratory species will likely require barriers to migration to be removed or mitigated as fragmentation of migration routes could lead to population declines [69] through road kill or fence entanglement [21]. Reduced movement and disruption of migration routes due to fences and roads are seen on many ungulate populations worldwide [21], [4], [64]. Preliminary studies investigating the effects of traffic on pronghorn proved unsuccessful due to traffic counters being destroyed by humans, but the effects of roads on migration nonetheless warrants future study. If fences cannot be completely removed, replacing barbed wire with smooth wire, lowering the fence height, raising the lowest wire strand and installing wildlife road-crossing structures could help prevent further range contraction of sensitive ungulate populations [18], [21], [2], [15].

Seasonal ungulate migrations are declining globally [3], [4], and of particular concern are those in temperate grasslands near the edge of their range [15], [13]. Our results should be used as a first step to implementing conservation and management efforts for this pronghorn population and to inform conservation of similar ungulate migrations in relatively open prairie regions. The methods we used can be used in both fragmented, highly developed landscapes to identify remaining corridors and bottlenecks as well as in natural landscapes to document previously unidentified corridors. We suggest prioritizing conservation efforts such as fence-mitigation measures and road-crossing structures in those areas used during both spring and fall migrations in the 1–5% corridor areas.

## Supporting Information

Figure S1 **Corridors created using the 1% threshold.** Least-cost (black and white line) and circuit theory (solid black) corridors connecting the habitat patches (dark gray outlines) at the 1% threshold on the Maxent and Analytic Hierarchy Process resistance surfaces for the fall (A) and (B), respectively, and the spring (C) and (D). Pronghorn locations are shown in white.
(TIF)

Figure S2 **Corridors created using the 5% threshold.** Least-cost (black and white line) and circuit theory corridors (solid black) connecting the habitat patches (black outlines) at the 5% threshold on the Maxent and Analytic Hierarchy Process resistance surfaces for the fall (A) and (B), respectively, and the spring (C) and (D). Pronghorn locations are shown in white.
(TIF)

Figure S3 **Corridors created using the 10% threshold.** Least-cost (black and white line) and circuit theory corridors (solid black) connecting the habitat patches (black outlines) at the 10% threshold on the Maxent and Analytic Hierarchy Process resistance surfaces for the fall (A) and (B), respectively, and the spring (C) and (D). Pronghorn locations are shown in white.
(TIF)

Figure S4 **Corridors created using the 15% threshold.** Least-cost (black and white line) and circuit theory corridors (solid black) connecting the habitat patches (dark gray outlines) at the 15% threshold on the Maxent and Analytic Hierarchy Process resistance surfaces for the fall (A) and (B), respectively, and the spring (C) and (D). Pronghorn locations are shown in white.
(TIF)

Figure S5 **Corridors created using the 20% threshold.** Least-cost (black and white line) and circuit theory corridors (solid black) connecting the habitat patches (dark gray outlines) at the 20% threshold on the Maxent and Analytic Hierarchy Process resistance surfaces for the fall (A) and (B), respectively, and the spring (C) and (D). Pronghorn locations are shown in white.
(TIF)

Table S1 **Area and percent of total area covered by land ownership types within northern Blaine, within the Montana portion of the study area where pronghorn migration corridors were identified.**
(DOCX)

Table S2 **Area and percent of total area covered by land cover classes within Montana and south-central Saskatchewan where pronghorn migration corridors were identified.**
(DOCX)

Table S3 **Percent of individual pronghorn locations falling within Analytic Hierarchy Process–least-cost path corridors during spring migration.**
(DOCX)

Table S4 **Percent of individual pronghorn locations falling within Analytic Hierarchy Process–Circuitscape corridors during spring migration.**
(DOCX)

Table S5 **Percent of individual pronghorn locations falling within Maxent–least-cost path corridors during spring migration.**
(DOCX)

Table S6 **Percent of individual pronghorn locations falling within Maxent–Circuitscape corridors during spring migration.**
(DOCX)

Table S7 **Percent of individual pronghorn locations falling within Analytic Hierarchy Process–least-cost path corridors during fall migration.**
(DOCX)

Table S8 **Percent of individual pronghorn locations falling within Analytic Hierarchy Process–Circuitscape corridors during fall migration.**
(DOCX)

Table S9 **Percent of individual pronghorn locations falling within Maxent–least-cost path corridors during fall migration.**
(DOCX)

Table S10 **Percent of individual pronghorn locations falling within Maxent–Circuitscape corridors during fall migration.**
(DOCX)

Text S1 **Environmental Variable Data Preparation.**
(DOC)

## References

[1] Berger J (2004) The last mile: how to sustain long-distance migration in mammals. Conserv Biol 18: 320–331.

[2] Bolger DT, Newmark WD, Morrison TA, Doak DF (2008) The need for integrative approaches to understand and conserve migratory ungulates. Ecol Lett 11: 63–77. 17897327 10.1111/j.1461-0248.2007.01109.x

[3] Wilcove DS, Wikelski M (2008) Going, going, gone: is animal migration disappearing? PLoS Biol 6: 1361–1364. 10.1371/journal.pbio.0060188 PMC2486312 18666834

[4] Harris G, Thirgood S, Hopcraft JGC, Cromsigt JPGM, Berger J (2009) Global decline in aggregated migrations of large terrestrial mammals. Endang Sp Res 7: 55–76.

[5] Bowlin MS, Bisson IA, Shamoun-Baranes J, Reichard JD, Sapir N, et al (2010) Grand challenges in migration biology. Integr Comp Biol 50: 261–279. 10.1093/icb/icq013 PMC7108598 21558203

[6] Sanderson FJ, Donald PF, Pain DJ, Burfield IJ, van Bommel FPJ (2006) Long-term population declines in Afro-Palearcic migrant birds. Biol Conserv 131: 93–105.

[7] Caudill CC, Daigle WR, Keefer ML, Boggs CT, Jepson MA, et al (2007) Slow dam passage in adult Columbia River salmonids associated with unsuccessful migration: delayed negative effects of passage obstacles or condition-dependent mortality? Can J Fish Aquat Sci 64: 979–995.

[8] Keefer ML, Peery CA, Daigle WR, Jepson MA, Lee SR, et al (2005) Escapement, harvest, and unknown loss of radio-tagged adult salmonids in the Columbia River-Snake River hydrosystem. Can J Fish Aquat Sci 62: 930–949.

[9] Dingle H, Drake VA (2007) What is Migration? Bioscience 57: 113–121.

[10] Fryxell JM, Sinclair ARE (1988) Causes and consequences of migration by large herbivores. Trends Ecol Evol 3: 237–241. 21227239 10.1016/0169-5347(88)90166-8

[11] Hebblewhite M, Merrill E, McDermid G (2008) A multi-scale test of the forage maturation hypothesis in a partially migratory ungulate population. Ecol Monogr 78: 141–166.

[12] Sawyer H, Kauffman MJ, Nielson RM, Horne JS (2009) Identifying and prioritizing ungulate migration routes for landscape level conservation. Ecol Appl 19: 2016–2025. 20014575 10.1890/08-2034.1

[13] Hannah L, Carr JL, Lankerani A (1995) Human disturbance and natural habitat: a biome level analysis of a global data set. Biodivers and Conserv 4: 128–155.

[14] Chape S, Blyth S, Fish L, Fax P, Spalding M (2003) United Nations List of Protected Areas. IUCN, Gland, Switzerland and Cambridge, UK and UNEO-WCMC, Cambridge, UK.

[15] Yackulic CB, Sanderson EW, Uriarte M (2011) Anthropogenic and environmental drivers of modern range loss in large mammals. Proc Natl Acad Sci USA 108: 4024–4029. 21368120 10.1073/pnas.1015097108 PMC3054034

[16] Brown JH (1984) On the relationship between abundance and distribution of species. Am Nat 124: 255–279.

[17] Channell R, Lomolino MV (2000) Trajectories to extinction: Spatial dynamics of the contraction of geographical ranges. J Biogeogr 27: 169–179.

[18] Laliberte AS, Ripple WJ (2004) Range contractions of North American carnivores and ungulates. Bioscience 54: 123–138.

[19] Smith DG, Hoppe TA (2000) Prairie Agricultural Landscapes: A Land Resource Review. Saskatchewan, Canada: Prairie Farm Rehabilitation Administration.

[20] Riley JL, Green SE, Brodribb KE (2007) A Conservation Blueprint for Canada's Prairies and Parklands. Saskatchewan, Canada: Nature Conservancy Canada.

[21] Harrington JL, Conover MR (2006) Characteristics of ungulate behavior and mortality associated with wire fences. Wildlife Soc B 34: 1295–1305.

[22] Beckmann JP, Siedler RG (2009) Wildlife and energy development: pronghorn of the Upper Green River Basin – year 4 summary. New York: Wildlife Conservation Society.

[23] Vander Wal E, Rodgers AR (2009) Designating seasonality using rate of movement. J Wildlife Manage 73: 1189–1196.

[24] Kolar JL, Millspaugh JJ, Stillings BA (2011) Migration patterns of pronghorn in southwestern North Dakota. J Wildlife Manage 75: 198–203.

[25] Yoakum JD, O'Gara BW, Howard VW (1996) Pronghorn on western rangelands. In Kausman PR, editor. Rangeland Wildlife. Denver, Colorado: Society for Range Management. pp 211–216.

[26] Beale DM, Smith AD (1970) Forage use, water consumption and productivity of pronghorn antelope in western Utah. J Wildlife Manage 34: 570–582.

[27] Pettorelli N, Vik JO, Mysteurd A, Gaillard J, Tucker CJ, et al (2005) Using the satellite-derived NDVI to assess ecological responses to environmental change. Trends Ecol Evol 20: 503–510. 16701427 10.1016/j.tree.2005.05.011

[28] Hamel S, Garel M, Festa-Bianchet M, Gaillard J-M, Cote SD (2009) Spring Normalized Difference Vegetation Index (NDVI) predicts annual variation in timing of peak fæcal crude protein in mountain ungulates. J Appl Ecol 46: 582–589.

[29] Hervert JJ, Bright JL, Henry RS, Piest LA, Brown MT (2005) Home-range and habitat-use patterns of Sonoran pronghorn in Arizona. Wildlife Soc B 33: 8–15.

[30] Martinka CJ (1967) Mortality of northern Montana pronghorns in a severe winter. J Wildlife Manage 31: 159–164.

[31] Barrett MW (1982) Distribution, behavior, and mortality of pronghorns during a severe winter in Alberta. J Wildlife Manage 46: 991–1002.

[32] Clary WP, Beale DM (1983) Pronghorn reactions to winter sheep grazing, plant communities, and topography in the Great Basin. J Range Manage 36: 749–752.

[33] Beckmann JP, Murray K, Seidler RG, Berger J (2012) Human-mediated shifts in animal habitat use: sequential changes in pronghorn use of a natural gas field in Greater Yellowstone. Biol Conserv 147: 222–233.

[34] Gavin SD, Komers PE (2006) Do pronghorn (*Antilocapra americana*) perceive roads as predation risk? Can J Zoolog 84: 1775–1780.

[35] Spillet JJ, Low JB, Sill D (1967) Livestock fences – how they influence pronghorn antelope movements. Logan, Utah: Utah State University Agricultural Experiment Station.

[36] Phillips SJ, Anderson RP, Schapire RE (2006) Maximum entropy modeling of species geographic distributions. Ecol Model 190: 231–259.

[37] Austin M (2007) Species distribution models and ecological theory: a critical assessment and some possible new approaches. Ecol Model 200: 1–19.

[38] Wikramanayake E, McKnight M, Dinerstein E, Joshi A, Gurung B, et al (2004) Designing a conservation landscape for tigers in human-dominated environments. Conserv Biol 18: 839–844.

[39] Beier P, Majka DR, Spencer WD (2008) Forks in the roads: choices in procedures for designing wild land linkages. Conserv Biol 22: 836–851. 18544090 10.1111/j.1523-1739.2008.00942.x

[40] Luan XF, Qu Y, Li DQ, Liu SR, Wang XL, et al (2011) Habitat evaluation of wild Amur tiger (*Panthera tigris altaica*) and conservation priority setting in north-east China. J Environ Manage 92: 31–42. 20828917 10.1016/j.jenvman.2010.08.001

[41] Phillips SJ, Dudik M, Schapire RE (2004) A maximum entropy approach to species distribution modeling. In Greiner R, Schuurmans D, technical coordinators. Proceedings of the International Machine Learning Conference. Alberta, Canada: ACM Press. pp 655–662.

[42] Elith J, Graham CH, Anderson RP, Dudik M, Ferrier S, et al (2006) Novel methods improve predictions of species' distributions from occurrence data. Ecography 29: 129–151.

[43] Hurley MV, Rapaport EK, Johnson CJ (2009) Utility of expert-based knowledge for predicting wildlife-vehicle collisions. J Wildlife Manage 73: 278–286.

[44] Clevenger AP, Wierzchowski J, Chruszcz B, Gunson KE (2002) GIS-generated, expert based models for identifying wildlife habitat linkages and planning mitigation passages. Conserv Biol 16: 503–514.

[45] Elith J, Phillips SJ, Hastie T, Dudik M, Chee YE, et al (2011) A statistical explanation of MaxEnt for ecologists. Divers Distrib 17: 43–57.

[46] Phillips SJ, Dudik M, Elith J, Graham CH, Lehman A, et al (2009) Sample selection bias and presence-only distribution models: implications for background and pseudo-absence data. Ecol Appl 19: 181–197. 19323182 10.1890/07-2153.1

[47] Fielding AH, Bell JF (1997) A review of methods for the assessment of prediction errors in conservation presence/absence models. Environ Conserv 24: 38–49.

[48] Guisan A, Zimmerman NE (2000) Predictive habitat distribution models in ecology. Ecol Model 135: 147–186.

[49] Phillips SJ, Dudik M (2008) Modeling of species distributions with Maxent: new extensions and a comprehensive evaluation. Ecography 31: 161–175.

[50] Saaty TL (1977) A scaling method for priorities in hierarchical structures. J Math Psychol 15: 234–281.

[51] Urban D, Keitt T (2001) Landscape connectivity: a graph-theoretic perspective. Ecology 82: 1205–1218.

[52] Urban DL, Minor ES, Treml EA, Schick RS (2009) Graph models of habitat mosaics. Ecol Lett 12: 260–273. 19161432 10.1111/j.1461-0248.2008.01271.x

[53] Adriaensen F, Cardon JP, deBlust G, Swinnen E, Villalba S, et al (2003) The application of ‘least-cost’ modeling as a functional landscape model. Landscape Urban Plan 64: 233–247.

[54] McRae BH (2006) Isolation by resistance. Evolution 60: 1551–1561. 17017056

[55] Theobald DM (2006) Exploring the functional connectivity of landscapes using landscape networks. In Crooks KR, Sanjayan M, editors. Connectivity Conservation. Cambridge, UK: Cambridge University Press. pp 416–443

[56] McRae BH, Beier P (2007) Circuit theory predicts gene flow in plant and animal populations. Proc Natl Acad Sci USA 104: 19885–19890. 18056641 10.1073/pnas.0706568104 PMC2148392

[57] Cushman SA, McKelvey KS, Schwartz MK (2008) Use of empirically derived source-destination models to map regional conservation corridors. Conserv Biol 23: 368–376. 19016821 10.1111/j.1523-1739.2008.01111.x

[58] McRae BH, Shah VB (2009) Circuitscape user's guide. Santa Barbara: The University of California. 12 p.

[59] McRae BH, Dickson BG, Keitt TH, Shah VB (2008) Using circuit theory to model connectivity in ecology, evolution, and conservation. Ecology 89: 2712–2724. 18959309 10.1890/07-1861.1

[60] Majka D, Jenness J, Beier P (2007) CorridorDesigner: ArcGIS tools for designing and evaluating corridors. Available at http://corridordesign.org.

[61] Owen-Smith N, Fryxell JM, Merrill EH (2010) Foraging theory upscaled: the behavioural ecology of herbivore movement. Philos T Soc B 365: 2267–2278. 10.1098/rstb.2010.0095 PMC2894968 20566503

[62] Sawyer H, Kauffman MJ (2011) Stopover ecology of a migratory ungulate. J Anim Ecol 80: 1078–1087. 21545586 10.1111/j.1365-2656.2011.01845.x

[63] Boccadori SJ, White PJ, Garrott RA, Borkowski JJ, Davis TL (2008) Yellowstone pronghorn alter resource selection after sagebrush decline. J Mammal 89: 1031–1040.

[64] Kaczensky P, Kuehn R, Lhagvasuren B, Pietsch S, Yang W, et al (2011) Connectivity of the Asiatic wild ass population in the Mongolian Gobi. Biol Conserv 144: 920–929. 21461051 10.1016/j.biocon.2010.12.013 PMC3040789

[65] Benhamou S (2011) Dynamic approach to space and habitat use based on biased random bridges. PLoS ONE 6: 14592. 10.1371/journal.pone.0014592 PMC3027622 21297869

[66] Horne JS, Garton EO, Krone SM, Lewis JS (2007) Analyzing animal movements using Brownian bridges. Ecology 88: 2354–2363. 17918412 10.1890/06-0957.1

[67] Prosser DJ, Cui P, Takekawa JY, Tang M, Hou Y, et al (2011) Wild bird migration across the Qinghai-Tibetan Plateau: A transmission route for highly pathogenic H5N1. PLoS ONE 6: 17622. 10.1371/journal.pone.0017622 PMC3052365 21408010

[68] Webster MS, Marra PP, Haig SM, Bensch S, Holmes RT (2002) Links between worlds: unraveling migratory connectivity. Trends Ecol Evol 17: 76–83.

[69] Hilty JA, Brooks C, Heaton E, Merenlender AM (2006) Forecasting the effect of land-use change on native and non-native mammalian predator distributions. Biodivers Conserv 15: 2853–2871.

