# Supplementary material for: Comparing Habitat Suitability and Connectivity Modeling Methods for Conserving Pronghorn Migrations
Source: PLoS One. 2012 Nov 16;7(11):e49390. doi: 10.1371/journal.pone.0049390 (PMC3500376; doi:10.1371/journal.pone.0049390)
Supplement: Table S1 — Area and percent of total area covered by land ownership types within northern Blaine, within the Montana portion of the study area where pronghorn migration corridors were identified. (DOCX) [file pone.0049390.s006.docx]

Table S1. Area and percent of total area covered by land ownership types within northern Blaine, within the Montana portion of the study area where pronghorn migration corridors were identified.

| Ownership Type | Area (km^2^) | Study Area (%) |
| --- | --- | --- |
| Local Government | 64 | 0.41 |
| State Government | 1,097 | 7.02 |
| Federal Government | 4582 | 29.36 |
| Tribal | 930 | 5.96 |
| Private | 8864 | 56.79 |
| Water | 70 | 0.45 |
| Undetermined | 0.01 | 0 |
| Total | 15,607 | 100 |
