# Supplementary material for: Comparing Habitat Suitability and Connectivity Modeling Methods for Conserving Pronghorn Migrations
Source: PLoS One. 2012 Nov 16;7(11):e49390. doi: 10.1371/journal.pone.0049390 (PMC3500376; doi:10.1371/journal.pone.0049390)
Supplement: Table S2 — Area and percent of total area covered by land cover classes within Montana and south-central Saskatchewan where pronghorn migration corridors were identified. (DOCX) [file pone.0049390.s007.docx]

Table S2. Area and percent of total area covered by land cover classes within Montana and south-central Saskatchewan where pronghorn migration corridors were identified.

| Land Cover Type | Area (km^2^) | Study Area (%) |
| --- | --- | --- |
| Agriculture | 7,009,118 | 26.45 |
| Developed | 180,727 | 0.68 |
| Exposed | 610,993 | 2.31 |
| Forest | 37,844 | 0.14 |
| Grassland | 14,904,794 | 56.27 |
| Pasture/Perennial Crop | 1,282,885 | 4.84 |
| Shrubland | 1,199,860 | 4.54 |
| Undefined | 43,829 | 0.16 |
| Water | 92,458 | 0.35 |
| Wetland | 1,127,831 | 4.26 |
| Total | 26,490,340 | 100 |
