# Supplementary material for: Comparing Habitat Suitability and Connectivity Modeling Methods for Conserving Pronghorn Migrations
Source: PLoS One. 2012 Nov 16;7(11):e49390. doi: 10.1371/journal.pone.0049390 (PMC3500376; doi:10.1371/journal.pone.0049390)
Supplement: Table S3 — Percent of individual pronghorn locations falling within Analytic Hierarchy Process–least-cost path corridors during spring migration. (DOCX) [file pone.0049390.s008.docx]

Table S3. Percent of individual pronghorn locations falling within Analytic Hierarchy Process–least-cost path corridors during spring migration.

Pronghorn Total Fix Count % in 1% % in 5% % in 10% % in 15% % in 20%

ID Corridor Corridor Corridor Corridor Corridor

123 442 17.87 53.17 70.14 71.72 71.95

128 910 1.32 80.55 90.55 100.00 100.00

129 239 3.35 98.33 100.00 100.00 100.00

130 287 21.25 44.25 63.41 94.43 96.52

134 144 2.78 90.28 100.00 100.00 100.00

135 216 50.46 71.30 100.00 100.00 100.00

136 132 11.36 78.79 100.00 100.00

137 959 12.41 24.19 24.19 24.19 24.30

138 124 20.16 63.71 66.13 66.94 73.39

140 179 1.12 45.81 55.87 58.66 88.83

141 378 0 28.84 51.59 82.80 83.07

142 191 0 71.73 74.35 84.29 85.86

145 164 0 0 0 0 0

108_380 415 2.17 46.75 91.08 99.52 100.00

110_690 92 48.91 59.78 60.84 68.48 100.00

113_648 89 10.11 56.18 100.00 100.00 100.00

118_580 79 11.96 56.80 73.42 79.47 83.76

Average 296.47 11.96 56.80 73.42 79.47 83.76
