# Supplementary material for: Comparing Habitat Suitability and Connectivity Modeling Methods for Conserving Pronghorn Migrations
Source: PLoS One. 2012 Nov 16;7(11):e49390. doi: 10.1371/journal.pone.0049390 (PMC3500376; doi:10.1371/journal.pone.0049390)
Supplement: Table S4 — Percent of individual pronghorn locations falling within Analytic Hierarchy Process–Circuitscape corridors during spring migration. (DOCX) [file pone.0049390.s009.docx]

Table S4. Percent of individual pronghorn locations falling within Analytic Hierarchy Process–Circuitscape corridors during spring migration.

Pronghorn Total Fix Count % in 1% % in 5% % in 10% % in 15% % in 20%

ID Corridor Corridor Corridor Corridor Corridor

123 442 28.28 28.73 68.33 71.49 71.49

128 910 12.31 18.57 62.53 100.00 100.00

129 239 54.39 75.31 100.00 100.00 100.00

130 287 60.98 61.67 86.41 94.43 95.82

134 144 0 18.75 93.75 100.00 100.00

135 216 46.30 46.76 57.87 100.00 100.00

136 132 30.30 51.52 79.55 100.00 100.00

137 959 10.22 18.14 23.46 24.30 24.40

138 124 24.19 27.42 66.94 71.77 71.77

140 179 45.25 48.60 53.63 84.36 90.50

141 378 55.82 65.87 77.25 80.95 80.95

142 191 40.84 47.12 75.92 81.68 88.48

145 164 0 0 0 0 0

108_380 415 50.60 100.00 100.00 100.00 100.00

110_690 92 11.96 59.78 63.04 93.48 100.00

113_648 89 37.08 75.28 96.63 100.00 100.00

118_580 79 13.92 34.18 81.01 100.00 100.00

Average 296.47 30.73 45.75 69.78 82.50 83.73
