# Supplementary material for: Comparing Habitat Suitability and Connectivity Modeling Methods for Conserving Pronghorn Migrations
Source: PLoS One. 2012 Nov 16;7(11):e49390. doi: 10.1371/journal.pone.0049390 (PMC3500376; doi:10.1371/journal.pone.0049390)
Supplement: Table S5 — Percent of individual pronghorn locations falling within Maxent–least-cost path corridors during spring migration. (DOCX) [file pone.0049390.s010.docx]

Table S5.Percent of individual pronghorn locations falling within Maxent–least-cost path corridors during spring migration.

Pronghorn Total Fix Count % in 1% % in 5% % in 10% % in 15% % in 20%

ID Corridor Corridor Corridor Corridor Corridor

123 442 9.95 50.00 70.36 71.72 71.72

128 910 1.54 83.96 99.78 100.00 100.00

129 239 28.03 100.00 100.00 100.00 100.00

130 287 4.18 31.71 47.74 92.68 94.77

134 144 29.86 99.31 100.00 100.00 100.00

135 216 2.31 68.98 100.00 100.00 100.00

136 132 28.79 98.48 100.00 100.00 100.00

137 959 6.36 23.77 24.19 24.30 24.30

138 124 16.94 50.00 6613 70.97 74.19

140 179 7.26 48.60 55.87 56.98 83.24

141 378 3.70 26.98 67.72 83.07 83.60

142 191 0 42.41 73.82 83.77 85.34

145 164 0 0 0 0 0

108_380 415 4.82 21.93 99.52 100.00 100.00

110_690 92 11.96 59.78 63.04 93.48 100.00

113_648 89 13.48 58.43 100.00 100.00 100.00

118_580 79 0 11.39 100.00 100.00 100.00

Average 296.47 9.95 51.51 74.60 81.00 83.36
