# Supplementary material for: Comparing Habitat Suitability and Connectivity Modeling Methods for Conserving Pronghorn Migrations
Source: PLoS One. 2012 Nov 16;7(11):e49390. doi: 10.1371/journal.pone.0049390 (PMC3500376; doi:10.1371/journal.pone.0049390)
Supplement: Table S7 — Percent of individual pronghorn locations falling within Analytic Hierarchy Process–least-cost path corridors during fall migration. (DOCX) [file pone.0049390.s012.docx]

Table S7. Percent of individual pronghorn locations falling within Analytic Hierarchy Process–least-cost path corridors during fall migration.

Pronghorn Total Fix Count % in 1% % in 5% % in 10% % in 15% % in 20%

ID Corridor Corridor Corridor Corridor Corridor

123 135 0 0 0 17.04 44.44

128 95 30.53 97.89 100.00 100.00 100.00

130 108 5.56 77.78 100.00 100.00 100.00

135 239 7.53 96.23 100.00 100.00 100.00

137 1022 0 .78 40.31 45.01 45.40

138 124 19.35 58.87 66.13 66.94 74.19

140 143 13.99 69.23 100.00 100.00 100.00

141 139 0 0 0 0 75.54

142 453 7.51 37.75 84.55 84.99 91.39

145 62 0 0 0 0 0

104_480 109 0 41.28 98.17 100.00 100.00

106_420 185 0 39.46 91.89 100.00 100.00

107_360 46 0 17.39 93.48 100.00 100.00

108_380 93 0 73.12 98.92 100.00 100.00

110_690 101 3.96 47.52 80.20 100.00 100.00

111_568 52 0 48.08 94.23 96.15 96.15

113_648 138 13.77 96.38 100.00 100.00 100.00

118_580 42 0 0 57.14 100.00 100.00

Average 182.56 5.68 44.54 72.50 78.34 84.84
