# Supplementary material for: Comparing Habitat Suitability and Connectivity Modeling Methods for Conserving Pronghorn Migrations
Source: PLoS One. 2012 Nov 16;7(11):e49390. doi: 10.1371/journal.pone.0049390 (PMC3500376; doi:10.1371/journal.pone.0049390)
Supplement: Table S9 — Percent of individual pronghorn locations falling within Maxent–least-cost path corridors during fall migration. (DOCX) [file pone.0049390.s014.docx]

Table S9. Percent of individual pronghorn locations falling within Maxent–least-cost path corridors during fall migration.

Pronghorn Total Fix Count % in 1% % in 5% % in 10% % in 15% % in 20%

ID Corridor Corridor Corridor Corridor Corridor

123 135 0 0 14.07 30.37 55.56

128 95 4.21 98.95 100.00 100.00 100.00

130 108 0 83.33 84.26 100.00 100.00

135 239 2.51 89.12 100.00 100.00 100.00

137 1022 0 26.42 44.81 45.01 45.21

138 124 3.23 41.94 66.94 72.58 73.39

140 143 46.85 60.84 88.11 100.00 100.00

141 139 0 0 0 52.52 84.89

142 453 26.49 35.54 44.81 85.21 88.08

145 62 0 0 0 0 0

104_480 109 9.17 77.06 98.17 100.00 100.00

106_420 185 71.35 91.89 92.43 100.00 100.00

107_360 46 67.39 100.00 100.00 100.00 100.00

108_380 93 26.88 79.57 100.00 100.00 100.00

110_690 101 0 61.39 75.25 88.12 100.00

111_568 52 0 46.15 94.23 96.15 96.15

113_648 138 4.35 84.78 100.00 100.00 100.00

118_580 42 0 7.14 80.95 100.00 100.00

Average 182.56 14.58 54.67 71.34 81.66 85.74
