# Supplementary material for: Comparing Habitat Suitability and Connectivity Modeling Methods for Conserving Pronghorn Migrations
Source: PLoS One. 2012 Nov 16;7(11):e49390. doi: 10.1371/journal.pone.0049390 (PMC3500376; doi:10.1371/journal.pone.0049390)
Supplement: Table S10 — Percent of individual pronghorn locations falling within Maxent–Circuitscape corridors during fall migration. (DOCX) [file pone.0049390.s015.docx]

Table S10. Percent of individual pronghorn locations falling within Maxent–Circuitscape corridors during fall migration.

Pronghorn Total Fix Count % in 1% % in 5% % in 10% % in 15% % in 20%

ID Corridor Corridor Corridor Corridor Corridor

123 135 8.89 13.33 14.81 18.52 39.26

128 95 63.16 78.95 93.68 93.68 93.68

130 108 76.85 79.63 99.07 99.07 99.07

135 239 77.41 79.08 85.36 88.70 92.05

137 1022 32.29 45.01 46.18 47.16 47.36

138 124 24.19 30.65 61.29 71.77 71.77

140 143 32.87 86.71 97.20 100.00 100.00

141 139 0 0 11.51 48.92 71.22

142 453 27.15 50.99 70.42 83.66 85.87

145 62 0 0 0 0 0

104_480 109 38.53 47.71 47.71 48.62 56.88

106_420 185 4.86 56.76 91.35 93.51 100.00

107_360 46 19.57 69.57 86.96 86.96 86.96

108_380 93 60.22 65.59 81.72 81.72 81.72

110_690 101 46.53 46.53 52.48 81.19 90.10

111_568 52 84.62 84.62 84.62 86.54 90.38

113_648 138 0 13.04 79.71 100.00 100.00

118_580 42 0 7.14 59.52 100.00 100.00

Average 182.56 3317 47.52 64.64 73.89 78.13
