# Supplementary material for: Comparing Habitat Suitability and Connectivity Modeling Methods for Conserving Pronghorn Migrations
Source: PLoS One. 2012 Nov 16;7(11):e49390. doi: 10.1371/journal.pone.0049390 (PMC3500376; doi:10.1371/journal.pone.0049390)
Supplement: Text S1 — Environmental Variable Data Preparation. (DOC) [file pone.0049390.s016.doc]

**SUPPLEMENTARY MATERIAL**

**Text S1. Environmental Variable Data Preparation**

*Distance from Water.—* Euclidean distance from water features of the 1:24,000 2000 National Hydrography Dataset Waterbody Features and the Atlas of Canada 1,000,000 National Frameworks Data, Hydrology – Drainage Network to each cell within the study area was used in the habitat suitability model (HSM). For the spring, all water bodies were included and assuming less water would be available in the fall, we excluded the ephemeral and temporary water bodies from these models by selecting water bodies designated in the attribute table as permanent water bodies.

*Land Cover.—* Land cover of the Northern Sagebrush Steppe region was provided by Northern Sagebrush Steppe Initiative (NSSI) partners (Alberta Sustainable Resource Development, Saskatchewan Ministry of Environment, Montana Fish, Wildlife & Parks) and created from the National Gap Analysis Program Land Cover Dataset for Montana and the 2000 Land Cover for Agricultural Regions of Canada national land cover dataset for Saskatchewan. Because very little forest was found in the study area, forest categories were combined in the AHP rankings (Beier et al. 2008).

*Normalized Difference Vegetation Index (NDVI).—* In the normalized difference vegetation index the scale ranges from -1 to 1, where -1 indicates a lack of vegetation and 1 indicates greenness saturation. We used 16 day composite vegetation indices images from NASA’s moderate resolution imaging spectrometer (MODIS) aboard the EOS-Terra satellite (Huete et al.1999). Images spanning the entire migration period (February 18 – April 30^th^ for spring 2008, October 11^th^ – January 7^th^ for fall 2008, March 11^th^ – June 6^th^ for spring 2009 and September 28^th^ – January 16^th^ for fall 2009) were downloaded for both seasons. Thirteen 16 day composite images were averaged and included in the spring models and 16 images were averaged for the fall models.

*Topographic Position Index.—*The ASTER Global 30 meter digital elevation model (METI and NASA 2009) was used to create a topographic position index (TPI) raster to represent terrain (Guisan et al. 1999). Topographic position was calculated with a 200 cell (6,000 meter) circular moving window. Elevation of each cell was compared with the mean elevation within the window to determine the topographic position of the cell. The landscape was categorized into four terrain components: valley bottom, gentle/flat slopes, steep slopes and ridge tops and used in the habitat suitability models.

*Oil and Gas Wells.—* Oil and gas well data from jurisdictions involved in the Northern Sagebrush Steppe Initiative (NSSI) (Saskatchewan Ministry of Environment and Montana DNRS, Board of Natural Gas) was used. We identified active oil and gas wells and calculated the Euclidean distance from each active well to each cell within the study area for inclusion in the habitat models. The development that often accompanies oil and gas drilling contributes to the amount of fencing and roads on the landscape as well, thus compounding the negative effects of energy exploration on pronghorn movement (Berger et al. 2006).

*Fencing.—* Pronghorn typically move unabated until a fence is reached. As such, we used a binary measure of fence presence/absence. Fence location was modeled using private land ownership data provided by NSSI and the Montana Public Land Ownership dataset. We predicted fence presence based on parcel ownership, size and ownership adjacency using the following assumptions: (1) adjacent parcels with the same mailing address and the same public ownership type are not fenced; (2) public lands less than 640 acres are leased to the largest adjacent private parcel owner; (3) there is limited fencing on public lands; (4) state lands and private lands smaller than 40 acres within tribal lands are not fenced; and (5) fencing exists along township boundaries within large parcels; (M. Suitor, University of Calgary, personal communication). To model fence locations along roads, we assumed: (1) small roads are not fenced on public lands except on Bureau of Land Management lands; and (2) large roads are fenced on all lands. Private land ownership data was not available for Saskatchewan fence modeling but fences on public lands were combined with the modeled Montana fence data.

*Distance to Roads.—* The 2000 TIGER roads dataset, along with a BLM roads dataset were merged for the Montana portion of the study area. Roads from the Saskatchewan Enhanced SURN Dataset from 2004 were used for the Saskatchewan portion of the study area. We calculated Euclidean distance from roads to each cell within the study area for inclusion in the habitat models.

**REFERENCES**

1. Beier P, Majka DR, Spencer WD (2008) Forks in the roads: choices in procedures for

designing wild land linkages. Conserv Biol 22: 836-851.

2. Huete A, Justice C, van Leeuwen W (1999) MODIS vegetation index (MOD 13): algorithm

theoretical basis document version 3. Tucson, AZ: University of Arizona.

3. Guisan A, Zimmerman NE (2000) Predictive habitat distribution models in ecology. Ecol

Model 135: 147-186.

4. Berger J, Berger KM, Beckmann JP (2006) Wildlife and energy development: pronghorn of

the Upper Green River Basin – year 1 summary. New York: Wildlife Conservation Society.
